# Exploring the Relationship Between Physical Activity and ICF Domains in Young Adults with Cerebral Palsy: A Comparison of Unilateral and Bilateral Cases

**DOI:** 10.3390/jcm15062391

**Published:** 2026-03-20

**Authors:** Lena Carcreff, Anne Tabard-Fougère, Geraldo De Coulon, Stéphane Armand, Alice Bonnefoy-Mazure

**Affiliations:** 1 Department of Geriatric Medicine, Angers University Memory Clinic, Research Center on Autonomy and Longevity, and Department of Physical Medicine and Rehabilitation, Angers University Hospital, 49100 Angers, France; 2 Kinesiology Laboratory, Geneva University Hospitals and University of Geneva, 1205 Geneva, Switzerland; 3 Paediatric Orthopedic Unit, Department of Child and Teenagers, Geneva University Hospitals and University of Geneva, 1205 Geneva, Switzerland; 4 Centre of Research on Skeletal Muscle and Movement, Geneva University Hospitals and University of Geneva, 1205 Geneva, Switzerland

**Keywords:** clinical gait analysis, patient perception, physical activity, 6-min walking test

## Abstract

**Background/Objectives**: Youths with cerebral palsy (CP) have reduced levels of physical activity (PA) due to motor impairments and functional difficulties. Few studies have observed its link with various factors and none in young adults with CP. This study aimed to investigate the relationships between PA and various factors in young adults with CP, such as gait function, endurance, participation, and personal/environmental influences. **Methods**: Participants over 15 years old with CP who underwent Clinical Gait Analysis (CGA), the 6 min walk test, and wore an actimeter (ActiGraph GT3X+) for seven days were included. PA was assessed by daily step count (NbSteps/day). Explanatory factors included the Gait Profile Score (GPS), walking speed, subjective walking perception, joint pain, fatigue, 6 min walk distance, SF-36 questionnaire scores, and lifestyle habits. Correlations, univariate, and multivariate regression models were used for the analysis. **Results**: Forty-seven CP patients (28 males, 19 females, mean age 23.6 years) were included, with 82% classified as GMFCS I and 18% as GMFCS II. The average NbSteps/day was 5685. Significant correlations were found between NbSteps/day and subjective perception, pain, GMFCS level, and walking speed. Multivariate regression identified walking speed and physiotherapy (PT) sessions as significant predictors of PA. **Conclusions**: PA in young adults with CP is linked to walking speed, GMFCS level, joint pain, fatigue, and PT. No differences have been observed between patient unilateral or bilateral CP. However, individual behaviors vary and are not fully explained by linear regression analysis.

## 1. Introduction

Cerebral palsy (CP) comes from irreversible injury of the developing fetal or infant brain. Affecting about 2/1000 live births, it is the most frequent motor disorder in children [1]. People with CP demonstrate motor impairments which have a great impact on their gross motor functions and eventually on their quality of life [2]. In addition to low motor function, secondary conditions such as pain and fatigue can further accelerate the decline in mobility among this population [3].

Physical activity (PA) is undeniably essential for health and well-being. Nowadays, about 80% of adolescents are insufficiently active compared to WHO’s recommendations [4]. Young people with CP prove to be even more sedentary than the general population [5]. Although PA of any intensity is beneficial for health, Moderate-to-Vigorous PA (MVPA) is known to provide higher benefits [6]. Decreased levels of PA during youth contribute to a risk of weight gain, reduced muscle mass and strength, result in major health issues, and are predictive of their level of PA during adulthood [7].

Numerous studies have tried to find factors explaining PA levels in individuals with CP, using self-reported questionnaires [8,9] or activity monitors worn for several days [10]. The time spent in MVPA and the number of steps taken in a day are the most usual outcomes with activity monitors [11]. The Gross Motor Function Classification System (GMFCS) level was found to be directly associated with PA in most of the published works [3]. Motor capacity, often measured by the standardized gross motor function measure (GMFM), was found associated with PA but without strong evidence [12]. The 6 min walking test (6MWT) was not always found associated with PA [13]. More recently, studies have examined the link between PA and Clinical Gait Analysis (CGA) outcomes. Spatiotemporal parameters, the Gait Deviation Index (GDI) and some physical examination parameters were found to correlate only moderately with PA and not unanimously [14,15]. As far as adults with CP are concerned, Lennon et al. found that the GMFCS level, the GDI, employment and age were predictors of walking activity [16]. To summarize, all these studies have not allowed for reaching any consensus regarding the factors explaining PA and very few studies have been dedicated to adults with CP. There is a limited number of studies dedicated to adults with cerebral palsy, and no consensus exists regarding the factors explaining physical activity in this population [17,18].

Since 2001, the International Classification of Functioning disability and health (ICF) has been helping to describe the health condition of people with CP, considering not only the body structure and function but also activities and participation. These three domains are influenced by contextual environmental and personal factors. The ICF framework could help to identify the associated factors with PA. Chagas et al. have notably mapped the predominant outcomes according to the ICF to provide an overview of the development of children, adolescents, and young adults with CP [19].

The purpose of this study was thus to explore the relationships between PA and outcomes from the ICF domains in young adults with CP. We hypothesized that PA would be positively associated with gait function, endurance, and participation, and negatively associated with pain and fatigue. The secondary objective was to compare unilateral and bilateral CP regarding all these domains, with the expectation that individuals with unilateral CP would demonstrate higher PA levels due to less severe motor involvement.

## 2. Materials and Methods

### 2.1. Design

This prospective cross-sectional study was conducted at Geneva University Hospitals (Switzerland) between 1 January 2011 and 31 December 2023 and was approved by the local ethics committee (CER: 10-135). This study was conducted in accordance with the principles of the Declaration of Helsinki. All the participants or their legal guardians gave their written informed consent to participate.

### 2.2. Participants

**Figure 1:**
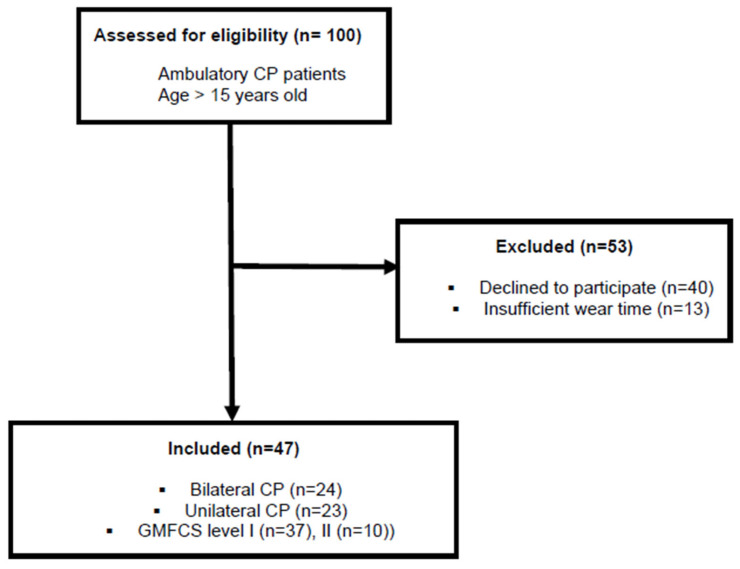
Flow chart of the inclusion and exclusion criteria leading to the selection of the study’s 47 cerebral palsy (CP) patients. Gross Motor Function Classification System (GMFCS); Clinical Gait Analysis (CGA).

Participants were recruited from patients scheduled for a Clinical Gait Analysis (CGA) in our laboratory. They were included if they met the following inclusion criteria: (1) a confirmed diagnosis of CP based on clinical symptoms and medical imaging; (2) ability to walk independently (with or without assistive devices), corresponding to GMFCS levels I–II; (3) age 15 years or older; (4) agreement to wear an ActiGraph GT3X+ activity monitor at sacrum level for 7 consecutive days. Exclusion criteria were recent orthopedic surgery or botulinum toxin injections within the past six months, as these could temporarily affect gait and physical activity. Figure 1 presents the flow diagram of participant selection.

### 2.3. Outcomes

**Figure 2:**
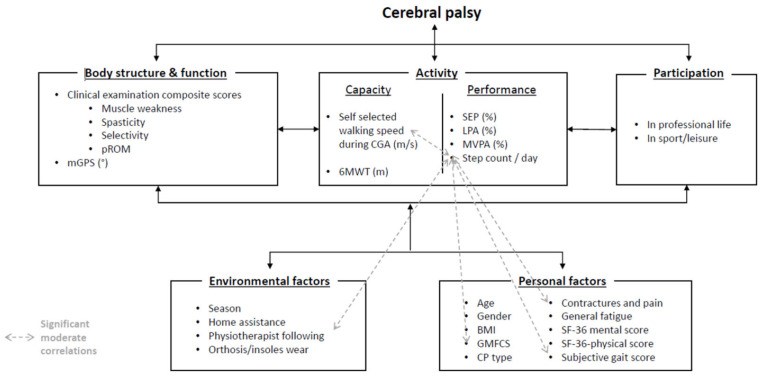
Illustration of the various parameters analyzed in the study according to the International Classification of Functioning disability and health. Modified Gait Profile Score (mGPS); passive range of motion (pROM); 6 min walking test (6MWT); Sedentary Physical Activity (SEP); Light-Intensity Physical Activity (LPA); Moderate-to-Vigorous Physical Activity (MVPA); Body Mass Index (BMI); Gross Motor Function Classification System (GMFCS); Cerebral palsy type (CP type).

Each ICF domain has been evaluated through one or several outcomes. The classification in the appropriate ICF domain was based on WHO’s recommendations as well as related published works [19]. An illustrated overview of the outcomes is provided in Figure 2. The following paragraphs detail the materials and methods to compute these outcomes.

#### 2.3.1. Clinical Examination Outcomes

Prior to CGA, a standardized clinical examination was conducted to the lower limbs including spasticity, selectivity, muscle weakness and passive range of motion (pROM). Muscle weakness was assessed using the Manual Muscle Testing (MMT) ranging from 0 to 5. Muscle spasticity was measured using the Modified Ashworth Scale (MAS) on a scale of 0 to 4 [20]. Selective motor control was evaluated with the Selective Control Assessment of the Lower Extremity (SCALE) on a scale of 0 to 2. pRoM was measured using a goniometer to the nearest 5 degrees. Based on Papageorgious et al. (2019), individual joint scores (hip, knee and ankle) and impairment were calculated (except pROM score based on Tabard-Fougere et al. (2022)) and combined into a composite score [21,22]. The leg with the lowest global score was considered the most affected.

#### 2.3.2. Clinical Gait Analysis Outcomes

All participants underwent a CGA equipped with a 12-camera motion analysis system (Vicon Mx3+, Oxford, UK, 2008–2015, and Oqus 7+, Qualisys, Göteborg, Sweden, 2015–2021). Markers were placed on the lower limbs and pelvis according to the Conventional Gait Model [23]. Patients were asked to walk barefoot at their own pace along a 12 m walkway. Data on each patient was collected over at least five gait cycles. Five gait cycles were selected to ensure reliable spatiotemporal gait measurements while balancing data quality with patient tolerance, as the literature demonstrates that 3–7 cycles provide excellent reliability for mean gait parameters [24,25].

From the marker trajectories, typical CGA outcomes were used to objectively assess gait function such the self-selected walking speed and the modified Gait Profile Score (mGPS). mGPS corresponds to the GPS without the hip rotation joint angle outcome due to the low accuracy and repeatability of this specific outcome [26]. Visual 3D V2023.09.3 (C-Motion, Inc., Germantown, MA, USA), the open-source Biomechanical ToolKit package and Matlab R2012b (MathWorks, Natick, MA, USA) software were used for the computation of kinematics, data analysis, and gait score calculations.

#### 2.3.3. Six-Minute Walking Test

Following CGA, patients were asked to perform a 6MWT to assess their gait capacity [27]. The distance traveled during the test was reported. Rest periods were individualized based on patient needs, with seated breaks of 2–5 min provided upon request if fatigue occurred.

#### 2.3.4. Subjective Gait Perception

At the end of the visit, patients were asked, “On a scale from 0 (worst walk) to 10 (optimal walk), how would you describe your gait today?”. They answered using a 10-point visual analog scale (VAS) which enabled us to assess the self-perceived view of their current gait.

#### 2.3.5. General Fatigue

General fatigue was evaluated thanks to the General Fatigue Scale questionnaire including 9 questions about the influence of fatigue on: motivation, PA level, exercise, work and responsibility, personal relationships with friends, family and social life [28]. The final score is between 7 (no impact of fatigue on the patient’s daily life) and 63 (large impact of fatigue on the patient’s daily life).

#### 2.3.6. Contracture and Pain

The contracture and pain at muscle and joint levels were evaluated using three specific questions: (1) “Do you have muscular contractures?”; (2) “Do you suffer from muscle pain?”; (3) “Do you suffer from joint pain?”. For these three questions, the answers were: “Never” (0); “very rarely” (1); “about once a month” (2); “about once a week” (3), and “every day” (4). To address this issue, these outcomes were dichotomized into two categories for each question: no (0)/yes (1–4). These questions were asked at the end of the entire data collection.

#### 2.3.7. Quality of Life

The self-reported Short Form Health Survey (SF-36) was used to evaluate health-related quality of life (QoL) [29]. Its items measure 8 dimensions: physical function, limitations due to emotional problems, limitations due to physical problems, mental health, social function, vitality, bodily pain and general perception of health. From these, a Physical Component Summary (PCS), and a Mental Component Summary (MCS) were calculated. The SF-36 uses a 0–100 scale, where 0 indicates the lowest level of QoL and 100 indicates the highest level of health. This self-reported questionnaire was either completed independently or with the assistance of a parent or friend. The two summary scores (PCS and MCS) were reported in our analysis to consider both dimensions of the patient’s QoL.

#### 2.3.8. Physical Activity

Physical activity was monitored with an Actigraph GT3X+ tri-axial accelerometer (ActiGraph Corporation, Pensacola, FL, USA) for 7 days, worn at sacrum level. Accelerometer data was converted into 60 s and 15 s epochs, using the Actigraph proprietary software (Actilife v6.13.4), to match wear time algorithms and activity cut-point definitions respectively. Wear time was computed from Choi et al.’s algorithm [30]. Patients with insufficient time spent on wearing (<20% of overall wear and less than five calendar days) were excluded from the analysis. Step count per day (StepCount/day), time spent in sedentary PA (%SED), in Light-Intensity PA (%LPA) and Moderate-to-Vigorous PA (%MVPA) were also computed via Actilife using the customized GMFCS-specific thresholds as recommended by Trost et al. [31].

The season (winter, spring, summer or autumn) during which the activity monitor was worn was also reported, as seasonal variations in weather, daylight hours, and temperature have been shown to influence PA [32]. Accounting for season helps control for environmental confounders that may impact daily step counts independently of individual functional capacity.

#### 2.3.9. Others

Several other information regarding the patients’ habits and life characteristics were collected. They were asked if:

- They live alone (1), with parents (2), as a couple (3) or as a couple with children (4)
- They have personal home assistance (yes/no)
- They work or study on a full-time basis (1) or a part-time basis (2)
- They perform sport regularly (yes/no)
- They have regular physiotherapist (PT) sessions (yes/no)
- They wear orthosis or orthopedic insoles (yes/no)

### 2.4. Data Analysis

Patients’ characteristics and assessed outcomes were described as mean and 95% Confidence Interval (95% CI) for quantitative variables and percentages for qualitative variables. These values were reported for the whole cohort as well as for the unilateral and bilateral CP separately. Parametric tests were used since the data were normally distributed across the whole cohort.

Unilateral and bilateral CP were compared using unpaired *t*-tests for quantitative variables and Chi-squared tests for qualitative variables.

Spearman’s correlations were computed to assess associations between the entire cohort’s PA outcomes and all the other outcomes. Correlation coefficients of 0.0–0.30 were considered weak, 0.30–0.50 moderate, 0.50–0.70 good and >0.70 high [33].

To analyze the influence of categorial factors such as performing physiotherapy, participating in sport, wearing orthosis or orthopedic insoles, receiving personal assistance, and presence of pain (joints, muscles), unpaired *t*-tests were conducted to compare the means of StepCount/Day between the groups defined on these factors.

Univariate and multivariate regression models were performed to investigate the effects of all outcomes on patients’ PA levels. To limit the number of covariates in the multivariate regression model and, considering the relatively small sample size, outcomes with weak correlation (r < 0.30) were excluded from the multivariate model. In addition, to avoid the problem of collinearity between covariates, parameters associated with others (based on a stronger correlation) were not included in the multivariate regression model. To guide variable selection for the multivariate model while limiting the number of covariates given the relatively small sample size, a directed acyclic graph as illustrated in Figure S1 (Supplementary Materials) was constructed a priori based on clinical knowledge and the existing literature [34,35]. In this case, the choice of the representative parameter was based on clinical relevance and the literature. Statistical analyses were performed using STATA software, version 13.1 (StataCorp LP, College Station, TX, USA). Statistical significance was fixed at *p* < 0.05 (two-sided).

## 3. Results

### 3.1. Cohort Characteristics

Forty-seven patients with CP (59% men) with a mean age of 23.6 (SD 6.9) years, with 82% of GMFCS I and 18% of GMFCS II were included in the study (Figure 1). Fifty-one per cent were unilaterally spastic. The mean StepCount/day was 5685 (SD 2175). For the time spent in different PA intensities, the means were as follows: %SED: 0.84, (SD 0.09), %LPA: 0.13, (SD 0.09) and %MVPA: 0.04, (SD 0.07).

### 3.2. Comparison Between Unilateral and Bilateral CP

When comparing the types of CP, no significant differences were observed in physical activity outcomes (StepCount/day: unilateral CP = 5727 (SD 2190) vs. bilateral CP = 5610 (SD 2207), *p* = 0.688). However, a significant difference was found in the 6MWT distance, with higher distances achieved by individuals with unilateral CP compared to those with bilateral CP (distance in meters: unilateral CP = 601.4 (SD 159.0) vs. bilateral CP = 386.1 (SD 207.4), *p* < 0.05). Individuals with unilateral CP reported a significantly better subjective gait score and experienced notably less fatigue compared to those with bilateral CP (subjective gait score: unilateral CP = 8.1 (SD 1.4) vs. bilateral CP = 6.7 (SD 2.4), *p* < 0.05; general fatigue: unilateral CP = 28.7 (SD 10.9) vs. bilateral CP = 35.5 (SD 14.0), *p* < 0.05). Finally, unilateral CP was associated with better gait function, evidenced by higher walking speed and a lower mGPS, as detailed in Table 1.

### 3.3. Correlations with Physical Activity Measurements

Significant moderate positive correlations were found between StepCount/day and subjective gait score (r = 0.36, *p* = 0.03), as well as walking speed (r = 0.31, *p* = 0.03) (Figure 2). No significant correlations were found between %MVPA, %LPA, % SEP with other parameters. A significant difference was found in daily step count based on the presence of pain (no pain: 6231 (SD 2404) StepCount/day vs. pain: 4927 (1597) StepCount/day, *p* = 0.04) on the daily step count. Correlation analyses are presented for descriptive purposes to illustrate unadjusted relationships between variables prior to multivariable adjustment.

### 3.4. Univariate and Multiple Linear Regression Models

The variables introduced in the models to explain PA (StepCount/day) were age, BMI, joint pain (yes or no), subjective gait score, general fatigue, PT following (yes or no), SF-36 MCS and PCS, and walking speed. As shown in Table 2, GMFCS level, joint pain, subjective gait score, and walking speed were found to be associated with PA of patients with CP by univariate regression analyses. In the multivariate regression model, only PT followed (95% CI: 269; 2686; *p* = 0.018) and walking speed appeared to be significant contributing factors (95% CI: 337; 5437; *p* = 0.028). This model accounted for only 26% of the variance of the StepCount/day in patients with CP.

As it is summarizing Table S1 (Supplementary Materials), no major violations of regression assumptions were identified. These checks confirm that the regression results are robust and reliable.

## 4. Discussion

This study explored the levels of PA in young adults with CP and its potential associations with various factors within ICF domains. These factors included clinical measurements and objective gait scores (body structure and function), walking speed and 6MWT distance (capacity), participation in sports and professional life (participation), season, home assistance, PT sessions (environmental factors), and quality of life (QoL), general fatigue, GMFCS level, pain, and age (personal factors). The main findings indicated that performance in young adults with CP is moderately linked with some factors in the capacity, personal, and environmental domains, with no significant impact of CP type. However, the multilinear model revealed that only walking speed and receiving PT significantly explained the variation in PA. This finding is particularly noteworthy as it suggests that despite the complex interplay of body function, personal, and environmental factors, walking capacity and access to therapeutic support emerge as the primary drivers of actual physical activity performance in this population.

Several studies have analyzed PA levels in children with CP, showing a daily step count around 5500 steps/day, similar to this study’s mean daily step count of 5685 for the entire cohort [7,10,36]. This result aligns with the study by Salie et al. (2021), which found a mean daily step count of around 5680 in a cohort of 26 adolescents with CP (mean age: 17.8 years, GMFCS I to III) [37]. In our cohort, no differences in daily step count were found based on the type of CP (unilateral spastic: 5757 (2190) vs. 5610 (2207), *p* > 0.05). However, a significant difference was observed based on GMFCS level, with a daily step count of 6011 for GMFCS I compared to 4480 for GMFCS II-III (*p* = 0.03). This result is confirmed by correlations observed between lower PA and higher functional levels (GMFCS). For instance, Van Wely et al. found that in children with bilateral CP, higher GMFCS levels and older age were associated with lower PA levels, particularly on weekends, as evidenced by a decreased daily step count [38]. Moreover, pain is one of the most common factors impacting the daily activities and sleep of young adults and adults with CP [39]. Approximately 40% of adults with CP report experiencing regular pain, making it the most frequently reported somatic symptom throughout their lives. This pain significantly impacts motivation to move, mobility, depression, anxiety, and overall daily functioning [39]. Pain is often localized in the lower limbs due to chronic muscle spasticity, joint misalignment, and invasive or repetitive surgical procedures. Pain levels tend to increase with age with a significantly higher proportion of adults in the 30–39 years old age range (74.5%) compared to those in younger and older group age (*p* < 0.029), which in turn affects PA levels, limiting participation in daily activities [39]. This phenomenon has been observed in the present study, where higher pain levels were associated with lower PA levels. These findings are consistent with existing literature on PA in children with CP, which shows associations between lower PA levels and personal factors such as GMFCS and the presence of pain.

An interesting finding in this study was the relationship between PA and subjective gait scores reported by the patients. Patients with a positive perception of their gait quality had a higher daily step count, highlighting the importance of self-perception in their capacity and function during daily life. Additionally, a significant correlation was observed between PA levels and whether individuals received PT. Upon entering adulthood, patients with CP commonly have the desire to reduce or even stop following their physiotherapy regimen. However, PT, or at least regular training, is essential to maintain physical functions [40]. Patients receiving PT had higher PA levels, reflecting their efforts to maintain body function and mobility through specific rehabilitation sessions. This finding aligns with the positive self-image and motivation observed in patients with higher subjective gait scores.

In 2020 Guinet et al. found no associations between PA and body structure and function [14]. The level of passive range of motion, muscle weakness, and muscle spasticity did not correlate with daily step count in this cohort, likely due to the majority of patients having a GMFCS level of I, indicating a low clinical impact on body structure. Furthermore, similar to the study by Guinet et al., no relationships were found between PA and objective gait scores, except for walking speed [14]. Thus, patients with a higher walking speed have a higher daily step count reflecting the level of function and capacity to move during their daily life.

In summary, the correlations between performance (PA levels) and function outcomes were moderate and non-uniform, with a link to walking speed but not the GPS. Similar observations were made regarding performance and personal factors, with associations between PA and subjective gait scores, receiving PT, and joint pain. Surprisingly, no associations were found between performance (evaluated by the daily step count) and a part of capacity (evaluated by 6MWT (endurance)). The univariate and multivariable models indicated that only walking speed and PT could explain part of the PA level. This underscores the importance of staying active and, above all, staying motivated to move, while trying to maintain one’s function and take care of oneself in adulthood.

Several limitations should be considered. The first is the small cohort size, particularly when categorized by GMFCS level; however, this is consistent with comparable studies, which typically report total samples ranging from 22 to 71 participants with per-level sample sizes frequently in single digits [7,37]. Additionally, the sample may be biased as it only included patients capable of completing a CGA and agreed to wear an activity monitor for a week. Future research should compare these findings with a similar age group from the general asymptomatic population. This retrospective study did not allow for detailed characterization of PT (frequency, duration or treatment modality), nor did it include quantitative pain assessment such as visual analog scale. Future prospective studies should integrate these variables to better determine their impact on PA levels. Additionally, our study focused exclusively on kinematic analysis without kinetic (ground reaction forces, joint moments, joint powers) or electromyographic data. This represents a significant constraint, as kinematic parameters alone provide limited insight into the underlying neuromuscular control strategies and muscle dysfunction patterns that characterize cerebral palsy gait [41,42]. Furthermore, future studies applying non-linear approaches, such as permutation entropy [43] and statistical physics metrics [44], may capture complex gait dynamics beyond traditional linear measures and provide deeper insights into neuromuscular control in CP.

Finaly, given the cross-sectional design, the direction of the associations between walking speed and PA cannot be determined. It is possible that faster walking facilitates higher PA or, conversely, that higher overall activity and physical capacity contribute to faster gait. Future longitudinal studies are needed to clarify underlying mechanisms. Despite these limitations, this study’s strength lies in its combination of various parameters reflecting different ICF domains with objective measures of gait function (mGPS), capacity (walking speed/6MWT), and personal/environmental factors (GMFCS, subjective gait score, contractures, pain and PT sessions).

## 5. Conclusions

The present results are highly relevant as most research has focused on children and adolescents with CP, with less attention given to young adults transitioning into adulthood. In this cohort of young adults with CP, PT follow-up and walking speed were independently associated with daily step count in multivariable analysis. These findings suggest that functional mobility and engagement in rehabilitation are key determinants of physical activity in this population. Indeed, in the recent publication of Cornec et al. [40], it appears that factors independently associated with increased motor rehabilitation satisfaction were: management of pain thanks to PT, efficient care coordination, information and receiving treatment from PT. Further studies are needed to better understand additional factors contributing to variability in PA levels.

Greater understanding of the links between PA levels and these factors could help shape improved care and rehabilitation programs.

## Figures and Tables

**Table 1:** Patient cohort characteristics for the all the cohort and according to cerebral palsy type (unilateral or bilateral spastic).

	All CP Patients (*n* = 47)	Unilateral Spastic (*n* = 24)	Bilateral Spastic (*n* = 23)
**Patients’ characteristics**			
Sex (M/F)	28/19	14/10	10/13
Age (years), mean (SD)	23.6 (6.9)	23.7 (6.9)	23.3 (7.0)
BMI, mean (SD)	22.7 (4.3)	23.1 (4.4)	22.3 (4.2)
**GMFCS level,** *n* (%)			
Level I	37 (82)	**24 (100)**	**13 (59)**
Levels II–III	10 (18)	**0 (0)**	**10 (41)**
**Activity and participation**			
**Physical activity measurement,** mean (SD)			
**Step Count/Day**	5685 (2175)	5757 (2190)	5610 (2207)
**% SEP**	0.84 (0.09)	0.82 (0.10)	0.86 (0.07)
**% LPA**	0.13 (0.09)	0.15 (0.09)	0.11 (0.08)
**% MVPA**	0.04 (0.07)	0.03 (0.01)	0.03 (0.10)
**Season—Actigraph wear,** *n* (%)			
Spring	15 (33)	11 (46)	4 (18)
Summer	7 (14)	2 (8)	5 (23)
Autumn	9 (19)	6 (25)	3 (14)
Winter	16 (34)	5 (21)	10 (45)
**Gait capacity measurement,** mean (SD)			
6MWT Distance (m)	533.6 (212.1)	**601.4 (159.0)**	**386.1 (207.4)**
**Personal and environmental factors**			
Subjective gait score (VAS), mean (SD)	7.4 (2.0)	**8.1 (1.4)**	**6.7 (2.4)**
General Fatigue, mean (SD)	32.3 (12.9)	**28.7 (10.9)**	**35.6 (14.0)**
**Contracture and Pain (yes/no),** *n* (%)			
Muscles contractures	15 (32)/32 (68)	8 (33)/16 (67)	7 (32)/15 (68)
Muscles pain	16 (35)/30 (65)	9 (38)/15 (62)	8 (35)/14 (65)
Joints pain	27 (57)/20 (43)	15 (62)/9 (38)	11 (50)/11 (50)
**Quality of life,** mean (SD)			
SF-36, MCS	42.7 (14.4)	43.2 (13.6)	43.7 (15.5)
SF-36, PCS	52.2 (11.9)	55.1 (10.4)	48.9 (12.9)
**Family situation,** *n* (%)			
Living with parents	35 (74)	18 (75)	17 (74)
Living alone	9 (20)	4 (17)	5 (22)
Living as a couple	2 (4)	1 (4)	1 (4)
Living as couple with children	1 (2)	1 (4)	0 (0)
**Home assistance,** *n* (%)			
Yes	2 (4)	0 (0)	2 (9)
No	45 (96)	24 (100)	21 (91)
**Employment status,** *n* (%)			
Full-time	32 (68)	15 (63)	17 (74)
Part-time	15 (32)	9 (37)	6 (26)
**Sport,** *n* (%)			
Yes	34 (72)	18 (75)	16 (70)
No	13 (28)	6 (25)	7 (30)
**Physio,** *n* (%)			
Yes	21 (45)	9 (37)	12 (52)
No	26 (55)	15 (63)	11 (48)
**Orthosis/Sole,** *n* (%)			
Yes	10 (21)	4 (17)	6 (26)
No	37 (79)	20 (83)	17 (74)
**Body structure and function**			
Walking speed (m/s), mean (SD)	1.20 (0.26)	**1.26 (0.20)**	**1.14 (0.30)**
modified GPS (°), mean (SD)	8.1 (3.0)	**7.3 (1.3)**	**9.0 (3.9)**
modified GPS asymmetry (%), mean (SD)	16.1 (14.0)	**18.2 (16.4)**	**14.0 (11.1)**
**Clinical Examination—Composite score**			
Composite spasticity (0–16), mean (SD)	13.3 (2.9)	13.7 (2.5)	12.9 (3.4)
Composite weakness (0–30), mean (SD)	24.3 (4.9)	25.1 (3.5)	23.4 (6.1)
Composite selectivity (0–12)**,** mean (SD)	10.0 (2.6)	**10.7 (1.5)**	**9.2 (3.4)**
Composite pROM (0–6), mean (SD)	4.4 (1.8)	4.5 (1.7)	4.1 (1.9)

Bold signifies *p* < 0.05. Cerebral palsy (CP); Gross Motor Function Classification System (GMFCS); Body Mass Index (BMI); Visual analog scale (VAS); Percentage of Sedentary Physical Activity (% SEP); Percentage of Light-Intensity Physical Activity (% LPA); Percentage of Moderate-to-Vigorous Physical Activity (% MVPA); Quality of life (QoL); SF-36; Mental Score (MCS); Physical Score (PCS); Gait Profile Score (GPS); passive range of motion (pROM).

**Table 2:** Results from univariate and multiple linear regression models for analyzing the associations between step count/day and covariates: bilateral CP, GMFCS level, joint pain, subjective gait score, general fatigue, SF-36 MCS, SF-36 PCS, and walking speed.

		Step Count/Day					
		Univariate Model		Multivariate Model			
	β	95% CI	*p*-Value	β	95% CI	*p*-Value	Adjusted r2
**Predictor variables**							**0.26**
Bilateral CP	−146	−1438; 1145	0.820	N.U	-	-	
GMFCS II-III	−1530	−3041; −20	**0.047**	−1301	−2846; 244	0.097	
Joint Pain—Yes	−1283	−2532; −35	**0.044**	−737	−1933; 458	0.220	
Subjective gait score	332	22; 643	**0.036**	NU	-	-	
General fatigue	−50	−100; 1	0.055	NU	-	-	
Physio—Yes	1123	−131; 2378	0.078	1477	269; 2686	**0.018**	
QoL—SF-36							
SF-36 MCS	12	−31; 56	0.574	23	−18; 63	0.262	
SF-36 PCS	46	−7; 99	0.085	N.U	-	-	
Subscore Vitality	38	−1.6; 75	0.060	N.U	-	-	
Objective gait scores							
Walking Speed	2592	186; 4998	**0.035**	2887	337; 5437	**0.028**	

Standardized regression coefficient (β); adjusted r2 represents explained variance; Confidence Interval (CI); Not Used (NU); **Bold signifies *p* < 0.05**. Cerebral palsy (CP); Gross Motor Function Classification System (GMFCS); Quality of life (QoL); SF-36; Mental Score (MCS); Physical Score (PCS).

## Data Availability

The data supporting the findings of this study are openly available: https://yareta.unige.ch/archives/7eb87ba8-0969-4db5-a98d-4d12893272dd (accessed on 16 March 2026).

## Supplementary Materials

The following supporting information can be downloaded at: https://www.mdpi.com/article/10.3390/jcm15062391/s1, Figure S1: This Directed Acyclic Graph (DAG) illustrating hypothesized causal pathways for daily step count. Blue nodes represent exposure variables (GMFCS, joint pain, physiotherapy, SF-36 mental score), orange node represents the mediator (walking speed), and red node represents the outcome (daily step count). Arrows indicate the hypothesized direction of causality. This DAG guided variable selection for the multivariable regression model; Table S1: Assessment of linear regression assumptions (multivariable model, *n* = 47).

## Abbreviations

The following abbreviations are used in this manuscript:

CP	Cerebral Palsy
GMFCS	Gross Motor Function Classification System
PA	Physical Activity
MVPA	Moderate-to-Vigorous Physical Activity
LPA	Light-Intensity Physical Activity
6MWT	6-Minute Walking Test
GMFM	Gross Motor Function Measure
CGA	Clinical Gait Analysis
GDI	Gait Deviation Index
ICF	International Classification of Functioning disability and health
pROM	Passive range of motion
MMT	Manual Muscle Testing
MAS	Modified Ashworth Scale
SCALE	Selective Control Assessment of the Lower Extremity
mGPS	Modified Gait Profile Score
SF-36	Self-reported Short Form Health Survey
QoL	Quality of Life
PCS	Physical Component Summary
MCS	Mental Component Summary
SEP	Sedentary Physical Activity
