# Supplementary material for: Exploring the Relationship Between Physical Activity and ICF Domains in Young Adults with Cerebral Palsy: A Comparison of Unilateral and Bilateral Cases
Source: J Clin Med. 2026 Mar 20;15(6):2391. doi: 10.3390/jcm15062391 (PMC13026951; doi:10.3390/jcm15062391)
Supplement: Supplementary file 1 [file jcm-15-02391-s001.zip › jcm-4172864-supplementary.pdf]

Supplementary Materials:

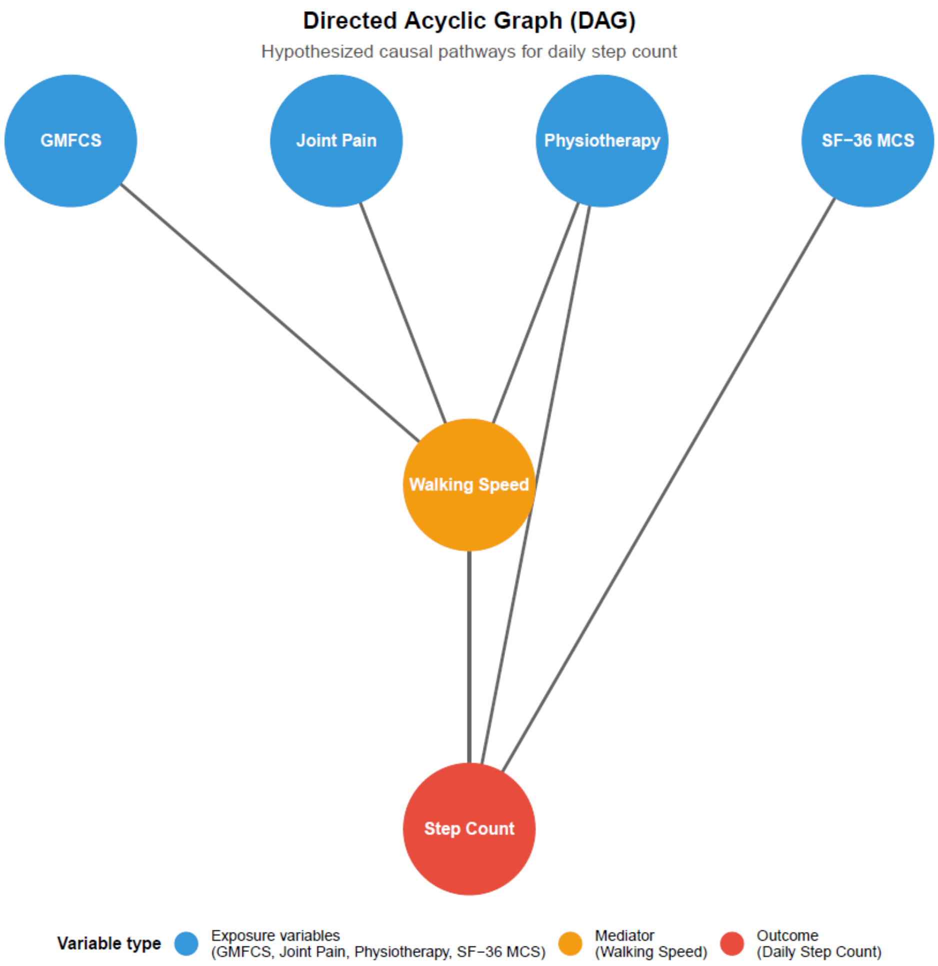

Figure S1. This Directed Acyclic Graph (DAG) illustrating hypothesized causal pathways for daily step count. Blue nodes represent exposure variables (GMFCS, joint pain, physiotherapy, SF-36 mental score), orange node represents the mediator (walking speed), and red node represents the outcome (daily step count). Arrows indicate the hypothesized direction of causality. This DAG guided variable selection for the multivariable regression model.

Table S1. Assessment of linear regression assumptions (multivariable model, n=47).

Assumption	Diagnostic method (Stata)	Statistical indicator	Results	Interpretation
**Linearity**	Residual vs fitted plot	Visual inspection	No systematic pattern	Assumption met
**Independence of errors**	Durbin-Watson test	1.93	Around 2	No autocorrelation
**Homoscedasticity**	Breusch-Pagan test	p=0.415	> 0.05	Constant variable
**Normality of residuals**	Q-Q plot Shapiro-Wilk	p= 0.744	> 0.05	Acceptable normality
**Multicollinearity**	Variance inflation Factor (VIF)	ranged from 1.04 to 1.29	< 5	No collinearity concern
**Influential observations**	Cook's distance	< 0.085	Below threshold	No influential outliers
